# Effects of mindfulness-based interventions on biomarkers in healthy and cancer populations: a systematic review

**DOI:** 10.1186/s12906-017-1638-y

**Published:** 2017-02-23

**Authors:** Kenji Sanada, Marta Alda Díez, Montserrat Salas Valero, María C. Pérez-Yus, Marcelo M. P. Demarzo, Jesús Montero-Marín, Mauro García-Toro, Javier García-Campayo

**Affiliations:** 1 Aragon Health Sciences Institute (IACS), Zaragoza, Spain; 2 Department of Psychiatry, Showa University School of Medicine, Tokyo, Japan; 3 The Primary Care Prevention and Health Promotion Research Network (REDIAPP), Barcelona, Spain; 4 Department of Psychiatry, Miguel Servet University Hospital, University of Zaragoza, Zaragoza, Spain; 5 Department of Psychology and Sociology, Faculty of Social and Human Sciences, University of Zaragoza, Teruel, Spain; 6 Department of Preventive Medicine, Federal University of Sao Paulo (UNIFESP), “Mente Aberta” - Brazilian Centre for Mindfulness and Health Promotion, Sao Paulo, Brazil; 7 Hospital Israelita Albert Einstein, Sao Paulo, Brazil; 8 Faculty of Health Sciences and Sports, University of Zaragoza, Huesca, Spain; 9 Research Institute of Health Sciences (IUNICS), University of Balearic Islands, Palma, Spain

**Keywords:** Mindfulness-based interventions, MBSR, Biomarkers, Cytokines, Interleukins, Neuropeptides, C-reactive protein

## Abstract

**Background:**

Only a small number of articles have investigated the relationship between mindfulness-based interventions (MBIs) and biomarkers. The aim of this systematic review was to study the effect of MBIs on specific biomarkers (cytokines, neuropeptides and C-reactive protein (CRP)) in both healthy subjects and cancer patients.

**Methods:**

A search was conducted using PubMed, EMBASE, PsycINFO and the Cochrane library between 1980 and September 2016.

**Results:**

A total of 13 studies with 1110 participants were included. In the healthy population, MBIs had no effect on cytokines, but were found to increase the levels of the neuropeptide insulin-like growth factor 1 (IGF-1). With respect to neuropeptide Y, despite the absence of post-intervention differences, MBIs may enhance recovery from stress. With regard to CRP, MBIs could be effective in lower Body Mass Index (BMI) individuals. In cancer patients, MBIs seem to have some effect on cytokine levels, although it was not possible to determine which specific cytokines were affected. One possibility is that MBIs might aid recovery of the immune system, increasing the production of interleukin (IL)-4 and decreasing interferon gamma (IFN-γ).

**Conclusions:**

MBIs may be involved in changes from a depressive/carcinogenic profile to a more normalized one. However, given the complexity and different contexts of the immune system, and the fact that this investigation is still in its preliminary stage, additional randomized controlled trials are needed to further establish the impact of MBI programmes on biomarkers in both clinical and non-clinical populations.

## Background

The word “mindfulness” is the translation of *sati* (Pali) or *smṛiti* (Sanskrit) into English. This is one of the most essential concepts in Buddhism and could be translated as “bare attention” or “present-centred awareness”, which is intended to mean a sort of non-judgemental, non-discursive attending to the here and now [1].

Mindfulness was first introduced into medical services and society by Kabat-Zinn in the 1970s [2]. He defined mindfulness as “paying attention in a particular way, on purpose, in the present moment, and non-judgementally”. The Mindfulness-Based Stress Reduction (MBSR) programme, a treatment protocol to administer mindfulness, was initially developed by Kabat-Zinn for patients with chronic pain [3]. This programme has since proved to be effective in treating not only healthy people under stress but also patients with various types of diseases: rheumatoid arthritis, ulcerative colitis, fibromyalgia, cancer, depression, post-traumatic stress disorder, schizophrenia, and many more [4–10]. Other treatment protocols using mindfulness have been developed that are based on MBSR but with specific psychoeducational components adapted to the target population. These protocols are described as Mindfulness-Based Interventions (MBIs) and include, for example, Mindfulness-Based Cognitive Therapy (MBCT), which is designed for major depression patients under high risk of relapse and recurrence [11].

A large number of articles on mindfulness have been published at a rapid rate in recent years. Williams and Kabat-Zinn [11] showed that the number of publications on mindfulness had reached 350 per year by 2010. Although there are probably over 700 studies at this point, only a small number of articles have investigated the relationship between MBIs and biomarkers both in healthy individuals and in patients with different disorders, despite their potential relevance. For instance, Matousek et al. [12] have reported the role of cortisol as a physiological marker of improvement with respect to MBSR. Moreover, there have only been two review studies related to cancer or HIV patients [13, 14].

### Aims

A systematic review was conducted with a specific focus on cytokines, neuropeptides and C-reactive protein (CRP) as biomarkers, because they are the most important and well-researched biomarkers of inflammatory parameters (except for cortisol, which has recently been reviewed in other work [15]), and further permitting comparisons to be made with previous studies. In addition, we classified samples into two categories: healthy individuals and cancer patients. Other disorders were not included in the review owing to the few studies available to date, based on a preliminary search. Thus, the aim of the present review was to provide a summary of the relationships between MBIs (MBSR is the most frequently used protocol but not the only one) and biomarkers (focused on cytokines, neuropeptides and CRP) both in healthy individuals and in cancer patients.

## Methods

We followed the PRISMA (Preferred Reporting Items for Systematic Reviews and Meta-Analyses) guidelines [16] and the recommendations of the Cochrane Collaboration [17]. The protocol was registered with PROSPERO (International Prospective Register of Systematic Reviews), with registration number CRD42016042302.

### Search

A search using PubMed, EMBASE, PsycINFO and the Cochrane library was conducted by an expert in this field (MSV). As an example, the searching strategy for the PubMed database was the following:

((“Mindfulness”[Mesh] OR mindfulness OR “mindfulness meditation” OR “meditation” OR “mindfulness based cognitive therapy” OR MBCT OR “mindfulness based stress reduction” OR MBSR)) AND (((((((“Cytokines”[Mesh]) OR “Interleukins”[Mesh]) OR “Biological Markers”[Mesh]) OR “Neuropeptides”[Mesh]) OR “C-Reactive Protein”[Mesh])) OR (((biomarker* OR cytokine* OR interleukin* OR neuropeptide* OR “C-reactive protein” OR CRP)))).

We included only studies published in English, French and Spanish between January 1980 and September 2016. The starting date was set because the first paper on MBIs was published in 1982 [3]. The literature search was conducted independently by two authors (KS and MCPY). Disagreements between the authors were solved by consensus, and when in doubt, the final decision was made in consultation with a third author (JGC). We followed standardized guidelines in order to enhance the quality of reporting in the present selective review. The last search was conducted on 04 October 2016.

### Inclusion criteria

Studies were required to fulfil the conditions described in the following sections [18].

#### Study designs

We included only the following experimental trials: randomized controlled trials (RCTs), non-randomized controlled trials (NRCTs), and open trials with a pre-post analysis.

#### Participants

We included only studies with healthy individuals or cancer patients. No restrictions were applied regarding the number of participants. However, trials with mixed types of participants (i.e. with cancer but also with other disorders) and studies of patients with disorders other than cancer were excluded. Examples of excluded trials include Fang et al. [19] and Malarkey et al. [20]. The former was conducted in patients with chronic pain but who also had depression and other disorders (not declared a diagnostic tool), whereas the latter was conducted in a workplace setting and most of the participants suffered from mild depression (nearly normal level), diagnosed according to the Center for Epidemiological Studies Depression (CES-D) that can measure depressive symptomatology; i.e. depressive cognitions, affect, and behaviours.

#### Biomarkers

We included only cytokines, neuropeptides and CRP as biomarkers because these were the most frequently studied biomarkers besides cortisol. Among the many studies investigating cortisol and MBIs, an independent paper on this biomarker has recently been published [15]. Where articles included biomarkers other than those targeted, they were included in the review, but only the findings related to the targeted biomarkers were described.

#### Interventions

The MBSR programmes included in this review were largely conducted in accordance with the standard programme developed by Kabat-Zinn at the University of Massachusetts Medical Center [2]. We also included curricula adapted from the standard MBSR programme, generally known as MBIs, such as MBCT and interventions involving Mindfulness Meditation (MM) [21], Mindfulness Training (MT) [22] and mindful awareness practices (MAPs) [23]. We included only those programmes with a minimum duration of 6 weeks because the effects of shorter protocols (known as low-dose interventions) should be studied independently owing to their own and different features. An example of this type of protocol can be found in Klatt et al. [24], and Creswell et al. [25].

#### Outcomes

Studies were judged eligible only if they assessed the relationship between MBIs and biomarkers. We excluded articles in which biomarkers were used as predictors to identify the participants likely to benefit from the intervention [26].

#### Accessibility of data

Only studies published as full papers were included.

### Assessment of study quality

Risk of bias in the different types of study designs was assessed with four criteria from the Cochrane Collaboration’s tool [27]: 1) adequate generation of allocation sequence; 2) concealment of allocation to conditions; 3) prevention of knowledge of the allocated intervention; and 4) dealing with incomplete outcome data. Studies that met three or more criteria were considered to be of high quality, and those that met fewer criteria were judged to be of low quality [28, 29]. Quality of interventions was evaluated according to three criteria [30]: use of a treatment manual, provision of therapy by specifically trained therapists, and verification of treatment integrity during the study. Two reviewers (MD and MCPY) independently assessed these criteria, and any discrepancies were discussed with a third reviewer (JGC) for consensus.

## Results

**Fig. 1:**
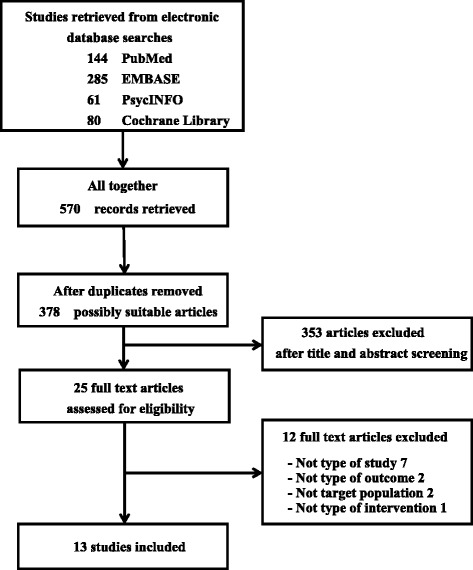
Algorithm of study selection (Following PRISMA guidelines)

The search yielded a total of 570 records (Fig. 1), of which 192 were duplicates. After screening the titles and abstracts, 25 articles were assessed as full text. We finally included 13 articles with a total of 1110 participants in this paper. One of these articles [31] was conducted as a 1-year follow-up study [32], and another two studies [33, 34] were conducted with the same population. We divided these trials into two categories based on the participant characteristics: healthy individuals and cancer patients.

The figure displays the details of the study search and selection process.

### Healthy individuals

We included seven articles [21, 22, 33–37] with a total of 750 healthy subjects. In most of these studies, the proportion of female participants ranged between 60 and 80%, except for one study [22], which was conducted on young male participants only. In two-thirds of the included studies, the average age was over 60 years. All of the studies were designed as RCTs, except one [22], which was NRCT. Regarding intervention type, six of the included studies were conducted with an 8-week intervention (MBSR or MT programmes) consisting of 2–2.5 h of weekly group sessions and 30–60 min of daily home practice. The remaining study [21] was implemented with a 6-week MM intervention based on a 1.5-h group session per week and daily home practice. We classified the seven included studies into the following two groups based on biomarkers.

#### Cytokines

We included a total of six articles utilizing cytokines as biomarkers. The characteristics of each study are shown in Table 1. Five cytokines were included: Interleukin (IL)-6, IL-8, tumour necrosis factor alpha (TNF-α) and interferon gamma (IFN-γ), as pro-inflammatory cytokines, and IL-10 as an anti-inflammatory cytokine. All of these studies evaluated the effects of MBSR or MM interventions on the above-mentioned cytokines. IL-6 was measured in three studies [21, 35, 36], IL-8 in two studies [33, 37], TNF-α in two studies [21, 37], and IFN-γ and IL-10 in one study [34].

**Table 1:** Cytokines in healthy individuals

Reference	Participants	Study design	Interventions	Controls	Follow up	Outcomes	Findings
Oken et al. (2010) [21]	31 Dementia caregivers	RCT (pilot)	MM (*N* = 10) 6 weeks + common first-week session	Education (*N* = 11) 6 weeks + common first-week session Respite (*N* = 10) 7 weeks respite care	No	IL-6, TNF-α, HS-CRP, salivary cortisol	There were no pre- and post-intervention differences in the levels of cytokines between the different intervention conditions (*p* > 0.05).
Barrett et al. (2012) [33]	149 ≥ 50y	RCT	MBSR (*N* = 51) 8 weeks	Exercise (*N* = 47) 8 weeks Observational Control (*N* = 51)	No	IL-8, neutrophil count, viral nucleic acid nasal secretion	Compared to the control group, IL-8 levels in the MBSR group were slightly higher in nasal wash collected during acute respiratory infection (*p* = 0.02).
Creswell et al. (2012) [35]	40 ≥ 55y	RCT (small)	MBSR (*N* = 20) 8 weeks	Wait-list Control (*N* = 20)	No	IL-6, CRP, pro-inflammatory gene expression	No significant treatment condition × time interaction for log-transformed IL-6 was observed (*p* = 0.57).
Gallegos et al. (2013) [36]	200 Community-dwelling ≥ 65y	RCT	MBSR (*N* = 100) 8 weeks	Wait-list Control (*N* = 100)	24 weeks	IL-6, IGF-1, IgM and IgG antibody response	There were no differences in the levels of IL-6 at post-MBSR intervention follow-ups. No associations were found between MBSR activities and IL-6 levels.
Rosenkranz et al. (2013) [37]	49 Community volunteers	RCT	MBSR (*N* = 33) 8 weeks	Health Enhancement Program (*N* = 16) 8 weeks	4 months	blister fluid TNF-α and IL-8, salivary cortisol	There were no pre- and post-intervention differences in the levels of TNF-α and IL-8 between the MBSR group and the active control intervention. Increased practice in the MBSR group was associated with a decrease in TNF-α levels, whereas the active control group tended to show the opposite relationship.
Hayney et al. (2014) [34]	149 ≥ 50y	RCT	MBSR (*N* = 51) 8 weeks	Exercise (*N* = 47) 8 weeks Observational Control (*N* = 51)	No	IFN-γ, IL-10 Serum influenza antibody, nasal IgA	There were no significant differences in the production of IFN-γ and IL-10 in the influenza-vaccinated individuals between the different intervention conditions.

*Abbreviations:* *HS-CRP* high-sensitivity C-reactive protein, *IFN-γ* interferon gamma, *IgA* immunoglobulin A, *IGF-1* insulin-like growth factor 1, *IgG* immunoglobulin G, *IgM* immunoglobulin M, *IL* interleukin, *MBSR* mindfulness-based stress reduction, *MM* Mindfulness Meditation intervention, *Open* open trial with a pre-post trial, *RCT* randomized controlled trial, *TNF-α* tumour necrosis factor alpha, *y* years

For IL-6, three trials revealed no significant effects of either MBSR or MM intervention, with no differences found pre- and post-intervention [21, 35] or during post-intervention follow-ups [32]. For IL-8, the results of the two trials revealed discrepancies. Barrett et al. [33] reported that the levels of IL-8 in nasal wash collected during acute respiratory infection in the MBSR group were slightly higher compared to those of the control group (*p* = 0.022). In contrast, Rosenkranz et al. [37] found no pre- and post-intervention differences in blister fluid levels of IL-8 between the MBSR group and the active comparison condition. For TNF-α, two trials [21, 37] found no apparent pre- and post-intervention differences in TNF-α levels between the different intervention conditions; however, Rosenkranz et al. [37] reported that additional practice in the MBSR group was associated with a decrease in TNF-α levels, whereas the active comparison group tended to show the opposite pattern. For IFN-γ and IL-10, Hayney et al. [34] showed that there were no significant differences between the intervention conditions in the production of these cytokines by peripheral blood mononuclear cells (PBMCs) in influenza-vaccinated individuals.

#### Neuropeptides and CRP

We included a total of four articles utilizing neuropeptides and CRP as biomarkers, three of which [21, 35, 36] were also included in the group that utilized cytokines. The characteristics of each study are shown in Table 2. In terms of neuropeptides, there was one study of insulin-like growth factor 1 (IGF-1) and neuropeptide Y (NPY) [22, 36], and two studies on CRP [21, 35].

**Table 2:** Neuropeptides and CRP in healthy individuals

Reference	Participants	Study design	Interventions	Controls	Follow up	Outcomes	Findings
Oken et al. (2010) [21]	31 Dementia caregivers	RCT (pilot)	MM (*N* = 10) 6 weeks + common first-week session	Education (*N* = 11) 6 weeks + common first-week session Respite (*N* = 10) 7 weeks respite care	No	IL-6, TNF-α, HS-CRP, salivary cortisol	There were no pre- and post-intervention differences in the levels of HS-CRP between the different intervention conditions (*p* > 0.05).
Creswell et al. (2012) [35]	40 ≥ 55y	RCT (small)	MBSR (*N* = 20) 8 weeks	Wait-list Control (*N* = 20)	No	IL-6, CRP, pro-inflammatory gene expression	Compared to the control group, a decrease in log-transformed CRP between pre- and post-intervention (*p* = 0.08).
Gallegos et al. (2013) [36]	200 ≥ 65y	RCT	MBSR (*N* = 100) 8 weeks	Wait-list Control (*N* = 100)	24 weeks	IL-6, IGF-1, IgM and IgG antibody response	There were positive effects of MBSR activities between IGF-1 levels and yoga, and sitting meditation (*p* < 0.01).
Johnson et al. (2014) [22]	281 Marine infantries	NRCT	MT (*N* = 147) 8 weeks	Usual training (*N* = 134)	No	plasma neuropeptide Y (NPY), norepinephrine	There were no significant pre- and post-intervention differences in plasma concentrations of NPY between MT intervention and the control group. The MT intervention group had lower plasma concentrations of NPY than the control group after the stressful training (*p* < 0.01).

*Abbreviations: HS-CRP* high-sensitivity C-reactive protein, *IGF-1*, insulin-like growth factor 1, *IgG* immunoglobulin G, *IgM* immunoglobulin M, *IL* interleukin, *MBSR* mindfulness-based stress reduction, *MM* Mindfulness meditation intervention, *MT* Mindfulness Training, *Open* open trial with a pre-post trial, *RCT* randomized controlled trial, *NRCT* non-randomized controlled trial, *TNF-α* tumour necrosis factor alpha

For IGF-1, increased practice of MBI activities overall was associated with significantly increased post-intervention IGF-1 production, specifically with yoga (*p* < 0.001) and sitting meditation (*p* < 0.01) [36]. For NPY, the MT intervention group had lower concentrations of this neuropeptide than the control group after the stress reduction training (*d* = 0.33; *p* < 0.01); however, there were no significant pre- and post-intervention differences in plasma concentrations of NPY between the MT intervention group and the control group [22].

For CRP, the two above-mentioned trials showed inconsistent findings. Creswell et al. [35] reported that the MBSR group had marginal decreases in log-transformed CRP compared to the control group (*p* = 0.075), with a pre- and post-intervention effect size of *d* = 0.88 in the MBSR group. In contrast, Oken et al. [21] found no significant pre- and post-intervention differences in the levels of CRP between the different intervention conditions (*p* = 0.891).

### Cancer patients

We included six articles (based on five studies) [23, 31, 32, 38–40], with a total of 360 participants. One of these, by Carlson et al. [31], was conducted as a 1-year follow-up study [32]. The characteristics of each study are shown in Table 3. The average age of the patients in these studies was in their 50s, with the exception of one study [23], where the mean age of the patients was in their 40s. The most frequent cancer type (total sample *n* = 304) was breast cancer (90.8%), which was followed by prostate cancer (3.9%). Of these five studies, three trials [23, 38, 40] recruited only breast cancer patients, although the other two studies [32, 39] enrolled patients with mixed types of cancer: breast and prostate cancers; and colon, breast, lung and prostate cancers, respectively. No similar criteria related to the duration of the cancer diagnosis were found in the included studies. Carlson et al. [32] included patients previously diagnosed with cancer for a median of 1.1 years, and similarly, Bower et al. [23] included patients diagnosed for a median of 4.0 years. There were trials in the other studies that did not clearly describe the duration of the cancer diagnosis, although Witek-Janusek et al. [38] recruited patients with “recently” diagnosed breast cancer. Lengacher et al. [40] enrolled breast cancer patients for whom a median time of 19 weeks had passed since treatment completion. Many of the patients in the selected articles had Stage I (44.4%) or Stage II (26.7%) cancer, while Lengacher et al. [39] enrolled only patients with Stage III (23.1%) or Stage IV (76.9%) cancer. Bower et al. [23] did not declare the details of cancer stage. There were differences in treatment regimen for the study populations of each trial. Witek-Janusek et al. [38] selected early-stage breast cancer patients who were treated with breast-conserving surgery and did not receive systemic chemotherapy. Lengacher et al. [40] examined whether the type of cancer treatment influenced the relationship between treatment completion and lymphocyte subset recovery. Bower et al. [23] included early-stage breast cancer patients who had completed local and/or adjuvant cancer therapy. The included articles consisted of the three study design types: open trials [31, 32, 39], RCTs [23, 40] and NRCTs [38].

**Table 3:** Cancer patients

Reference	Participants	Study design	Interventions	Controls	Follow up	Outcomes	Findings
Carlson et al. (2003) [32]	59 Early stage BC 49 PC 10	Open	MBSR (*N* = 59) 8 weeks	None	No	TNF, IFN-γ, IL-4, IL-10, NK, NKT, B, T total, T helper, and T cytotoxic cells	Although there were no significant differences in the overall number of lymphocytes or cell subsets, T cell production of IL-4 increased and IFN-γ decreased, whereas NK cell production of IL-10 decreased.
Carlson et al. (2007) [31]	59 Early stage BC 49 PC 10	Open	MBSR (*N* = 59) 8 weeks	None	12 months	TNF, IFN-γ, IL-4, IL-10, NK, NKT, B, T total, T helper, and T cytotoxic cells, salivary cortisol, blood pressure, heart rate	Immune patterns over the year indicated a continued reduction in Th1 (pro-inflammatory) cytokines. T-cell population of TNF, IFN-γ, and IL-4 decreased substantially over the course of the year between pre- and post-intervention, and across the follow-up assessments.
Witek-Janusek et al. (2008) [38]	96 Early stage BC 66 healthy 30	NRCT	MBSR (*N* = 38) 8 weeks	Control (usual care) (*N* = 28) 8 weeks Healthy Control (*N* = 30)	1 month	IL-2, IL-6, IFN-γ, IL-4, IL-10, T, NK, T helper, and T cytotoxic cells, NK cell activity, plasma cortisol	Although the non-MBSR group showed continued reduction in IFN- γ production with increased IL-4, IL-6, and IL-10 production between pre- and 1-month post-intervention, the MBSR group re-established their cytokine production levels over time.
Lengacher et al. (2012) [39]	52 Advanced stage cancer 26 caregivers 26	Open	MBSR (*N* = 52) 6 weeks	None	No	salivary IL-6 (2 times/day), salivary cortisol	There was a significant reduction in salivary IL-6 in all MBSR participants pre- to post-intervention (*p* = 0.002).
Lengacher et al. (2013) [40]	82 Early ~ advanced stage BC	RCT	MBSR (*N* = 40) 6 weeks	Control (usual care) (*N* = 42) 6 weeks	No	Th1: IFN-γ, Th2: IL-4, T, NK, and B cells	The MBSR group had T cells more readily activated by the mitogen PHA compared to the control group (*p* =0.002). The production of IFN-γ showed no significant changes between pre- and post-intervention; however, IL-4 production decreased compared to the control group. There was an increase in the Th1/Th2 ratio in the MBSR group (*p* = 0.03).
Bower et al. (2015) [23]	71 Early stage BC	RCT	MAPs (*N* = 39) 6 weeks	Wait-list Control (*N* = 32) 6 weeks	3 months	IL-6, CRP, 19 pro-inflammatory gene transcripts, NF-κB, anti-inflammatory glucocorticoid receptor (GR), CREB family factors, Type I interferon response factors, TNF receptor type II	The MAPS intervention group showed a significant decline in pro-inflammatory gene expression from baseline to post-intervention (*p* = 0.009). There were no significant effects of intervention for IL-6, CRP, and TNF receptor type II.

*Abbreviations: BC* breast cancer, *CREB* cAMP response element-binding protein, *IFN-γ* interferon gamma, *IL* interleukin, *MAPs* mindful awareness practices, *MBSR* mindfulness-based stress reduction, *NK* natural killer, *NKT* natural killer T, *Open* open trial with a pre-post trial, *PC* prostate cancer, *PHA* phytohemagglutinin, *RCT* randomized controlled trial, *NRCT* non-randomized controlled trial, *Th* T-helper, *TNF* tumour necrosis factor

With regard to the intervention characteristics, all of the included studies were conducted with a 6- or 8-week intervention involving MBSR or MAPs. Lengacher et al. [39, 40] adapted the intervention into a 6-week MBSR programme that included the entire content of the standard 8-week programme developed by Kabat-Zinn [2]. One of the studies [39] was conducted with group sessions consisting of three live sessions and three at-home practices for advanced-stage cancer patients. Bower et al. [23] used a 6-week MAP programme at UCLA (http://marc.ucla.edu). The other three studies [31, 32, 38] were conducted with an 8-week MBSR intervention providing 1.5–2.5 h of weekly group sessions in accordance with the original standard [2].

All of the selected studies evaluated the effects of MBSR or MAPs on cytokines and CRP as the biomarkers. Neuropeptides were not assessed. Four of the six trials examined the levels of both pro- and anti-inflammatory cytokines, while the remaining two trials [23, 39] evaluated IL-6 and CRP, and IL-6 levels, respectively.

In relation to pro-inflammatory cytokines, three cytokines were examined: IFN-γ, IL-6 and TNF. For IFN-γ, the findings of four trials [31, 32, 38, 40] were in disagreement. Although two trials showed that T cell production of IFN-γ decreased substantially compared to pre-intervention (*d* = 0.38; *p* < 0.01) [32], and across the 6- and 12-month follow-up (*d*_6_ = 0.94; *d*_12_ = 1.00; *p* < 0.001) [31], one trial [38] reported that the production of IFN-γ in women by PBMCs in the MBSR group increased significantly compared to that in the control group between the pre-intervention assessment and 1 month post-intervention (*p* = 0.027). Another trial [40] reported that phytohaemagglutinin-induced T cell production of IFN-γ in the MBSR group did not change significantly between pre- and post-intervention. For IL-6, Witek-Janusek et al. [38] found that production of IL-6 by PBMCs in the MBSR group was reduced with respect to the control group between the pre-assessment and 1 month post-intervention (*p* = 0.008). On the other hand, Lengacher et al. [39] reported that a significant overall reduction in salivary IL-6 was observed in both patients and caregivers from pre- to post-intervention. In a study performed by Bower et al. [23], there was no significant effect of the MAP intervention on IL-6 (*p =* 0.158). For TNF, only one trial [31] was conducted, which showed that T cell production of TNF decreased substantially between pre-intervention and both the 6- and 12-month follow-up (*d*_6_ = 0.92; *d*_12_ = 1.13; *p* < 0.001).

With respect to anti-inflammatory cytokines, two cytokines were examined: IL-4 and IL-10. For IL-4, the findings of four trials [31, 32, 38, 40] were in disagreement. Carlson et al. [31] reported that IL-4 production decreased between pre-intervention and the 6- and 12-month follow-up (*d*_6_ = 1.01; *d*_12_ = 1.37; *p* < 0.001). Similarly, two trials [38, 40] showed that IL-4 production in the MBSR group decreased compared to that in the control group between pre-assessment and 1 month post-intervention [38] or post-intervention [40]. However, the remaining trial [32] revealed that IL-4 production increased significantly between pre- and post-intervention (*p* < 0.01). For IL-10, two trials [32, 38] were conducted. Carlson et al. [32] found that natural killer (NK) cell production of IL-10 decreased between pre- and post-intervention (*d* = 0.33; *p* < 0.01), while Witek-Janusek et al. [38] showed that IL-10 production by PBMCs in the MBSR group decreased with respect to the control group between pre- and 1 month post-intervention (*p* < 0.035).

For CRP, only one study [23] reported that no significant effect was found from the MAP intervention on CRP (*p =* 0.415).

### Quality of included studies and interventions

With regard to risk of bias [27], seven studies were considered to be of high quality, and six were considered low quality (Table 4) [27]. In relation to the quality of the interventions, the use of a treatment manual was reported in all trials, therapist training in eight trials, and treatment integrity in none of the trials.

**Table 4:** Quality of included studies

Study	Trial quality^a^	Intervention quality
Oken et al. (2010) [21]	AS (+) AC (+) PK (+) IO (?)	Manual (+) Training (+) Integrity check (?)
Barrett et al. (2012) [33]	AS (+) AC (+) PK (-) IO (+)	Manual (+) Training (+) Integrity check (?)
Creswell et al. (2012) [35]	AS (+) AC (+) PK (-) IO (+)	Manual (+) Training (+) Integrity check (?)
Gallegos et al. (2013) [36]	AS (+) AC (+) PK (-) IO (+)	Manual (+) Training (?) Integrity check (?)
Rosenkranz et al. (2013) [37]	AS (+) AC (+) PK (+) IO (?)	Manual (+) Training (+) Integrity check (?)
Hayney et al. (2014) [34]	AS (+) AC (+) PK (+) IO (?)	Manual (+) Training (+) Integrity check (?)
Johnson et al. (2014) [22]	AS (+) AC (+) PK (-) IO (+)	Manual (+) Training (?) Integrity check (?)
Carlson et al. (2003) [31]	AS (-) AC (-) PK (-) IO (+)	Manual (+) Training (?) Integrity check (?)
Carlson et al. (2007) [32]	AS (-) AC (-) PK (-) IO (+)	Manual (+) Training (?) Integrity check (?)
Witek-Janusek et al. (2008) [38]	AS (-) AC (-) PK (-) IO (?)	Manual (+) Training (+) Integrity check (?)
Lengacher et al. (2012) [39]	AS (-) AC (-) PK (-) IO (?)	Manual (+) Training (+) Integrity check (?)
Lengacher et al. (2013) [40]	AS (+) AC (+) PK (-) IO (?)	Manual (+) Training (+) Integrity check (?)
Bower et al. (2015) [23]	AS (+) AC (+) PK (?) IO (-)	Manual (+) Training (?) Integrity check (?)

*AS* adequate generation of allocation sequence, *AC* concealment of allocation, *PK* prevention of knowledge of the allocated intervention, *IO* dealing with incomplete outcome data

^a^Risk of bias: low (+), high (–), or unclear (?) [24]

## Discussion

To our knowledge, this is the first systematic review providing a summary of the relationships between MBIs (MBSR and interventions involving MM, MT or MAPs) and biomarkers, with a focus on cytokines, neuropeptides and CRP, in both healthy subjects and cancer patients. We finally examined 12 articles (based on 13 studies) with a total of 1110 participants, which we divided into two categories: healthy individuals and cancer patients. None of the included studies could be considered to be of high quality, taking together into account the trial design, risk of bias and the quality of interventions [27].

### Healthy individuals

With respect to our seven selected articles (two studies were conducted with the same sample), we should consider the composition of participants and the characteristics of the interventions. First, there was a remarkable difference in the composition of men and women. The proportion of female participants was over 60% in many of the included studies. A second finding is the relatively high mean age of the subjects; more than half of the population were aged 60 years or older. Future trials with a more balanced composition of participants are needed. On the other hand, the interventions were conducted using mainly an 8-week programme (MBSR or MT) based on the standard design developed by Kabat-Zinn, with a 2–2.5-h group session per week, although one study was conducted with a 6-week MM intervention consisting of 1.5 h of weekly group sessions.

Our findings in healthy individuals indicate that the intervention programmes had no apparent effect on cytokines: of the six articles, three studies [21, 35, 36] investigated the levels of IL-6 without finding significant changes resulting from the interventions. Creswell et al. [35] noted that the levels of IL-6 at baseline evaluation were low because of the participants’ low risk of cardiovascular or inflammatory diseases. IL-6 is known to be one of the earliest and more important mediators of the induction of acute-phase protein synthesis [41], and it is an important regulator of CRP production by the liver [20]. Breines et al. [42] found that participants who were higher in self-compassion showed significantly lower IL-6 responses to acute psychosocial stressors. On the other hand, some studies have reported that IL-6 may have pro- or anti-inflammatory effects, depending on the cell or tissue context [43]. Given this, it seems difficult to evaluate the changes in IL-6 levels between pre- and post-intervention.

Similarly, the study by Hayney et al. [34] concluded that there were no significant differences in the production of IFN-γ and IL-10 under the intervention conditions, because it is more difficult to demonstrate improvements resulting from any therapeutic intervention in healthy individuals. Of these cytokines, IFN-γ is known to be a pro-inflammatory cytokine that is important for immunity against viral and intracellular bacterial infections [14], and it is mainly produced by T-helper (Th)1 and NK cells. IL-10 is known to be an anti-inflammatory cytokine that reduces the production of IFN-γ, and it is mainly produced by Th2 cells and monocytes.

The results for cytokines IL-8 and TNF-α were inconsistent. Of these cytokines, IL-8 is known to be a pro-inflammatory cytokine that promotes the activation and migration of neutrophils, and it is mainly produced by macrophages and monocytes, among others. TNF-α is known to be a typical pro-inflammatory cytokine that is involved in protection against infection, and for its anti-tumour effect. It is mainly produced by macrophages. Future trials should evaluate the effects of MBIs on cytokines using patients with different characteristics; for example, participants who are younger, of a different gender, or who have increased levels of CRP might be used.

With respect to neuropeptides and CRP, our findings revealed that the intervention programmes elicit a certain effect. For neuropeptides, the eligible studies included one with IGF-1 and another with NPY. Gallegos et al. [36] found that MBI activities, particularly yoga and sitting meditation, were associated with significantly higher levels of post-intervention IGF-1. IGF-1, also known as somatomedin C, is known to be a growth factor that mediates cell growth and development. As these techniques focus on breathing, emotional awareness, and cognitive moment-by-moment awareness [36], increased IGF-1 levels may therefore be related to the enhancement of cognitive function, and to cancer prevention. Levine et al. [44] demonstrated that an increased protein intake and the resulting increase in IGF-1 was associated with reduced cancer in older adults, whereas a low protein diet was likely to be useful for the prevention of cancer during middle age.

Johnson et al. [22] reported that the MT intervention had lower concentrations of NPY than the control group after stressful training, although NPY levels did not significantly differ between the groups, either at baseline or at post-intervention. NPY is known to be a peptide neurotransmitter that represents a protective factor in the face of stress [45]. Previous studies showed that higher levels of blood NPY in response to acute stress predicted better performance during military training [46], and less psychological distress [47]. In the study conducted by Johnson et al. [22], the above-mentioned finding suggests that MT intervention may have a beneficial effect, enhancing recovery from stress; i.e. responses to stress may have been improved through MT intervention. Finally, our findings for CRP indicated inconsistent effects. Interestingly, however, Malarkey et al. [20] reported a large body mass index (BMI) interaction effect for CRP, although the participants included were not only healthy individuals but also patients with mild depression, according to the CES-D diagnosis. Future trials should consider subjects with lower BMIs to better evaluate the effects of MBIs on CRP.

### Cancer patients

In cancer patients, our findings suggest that MBSR may have some effect on the levels of cytokines, although we could not determine which specific cytokines. This can be said to promote immune homeostasis more rapidly. Witek-Janusek et al. [38] reported that the reductions in Th2 cytokines (IL-4, IL-6 and IL-10) may allow for normalization of Th1 cytokines (e.g., IFN-γ), and Lengacher et al. [40] showed that MBSR may confer some beneficial effects on immune recovery. Another study [31] identified that the changes in the immune profiles, with increases in T cell production of IL-4, and decreases in IFN-γ, were consistent with a shift in the balance from a Th1 (pro-inflammatory) to a Th2 (anti-inflammatory) environment. An increase in IFN-γ has often been observed in subjects with depression [48], and increases in IFN-γ and other TH1 type cytokines are associated with pro-inflammatory effects. The cytokine pattern of depression has been comparable to that of cancer [48]. On the other hand, IL-4 is known to be an anti-inflammatory cytokine that promotes Th2 cell growth and differentiation, and it is mainly produced by activated Th2 cells, mast cells and natural killer T cells, among others. Therefore, it seems to indicate that the immune changes might show a shift away from a depressive/carcinogenic cytokine profile to a more normalized one.

With regard to the conditions of the patients and the characteristics of the interventions in our six selected articles (based on five studies), no differences could be found in the mean age of patients in any of the studies except one, and many of the patients were in their 50s, on average. In relation to the different cancers, the most frequent type was breast cancer (90.8%). Of the included five studies, three trials [23, 38, 40] were conducted with only breast cancer patients; however, the other two [32, 39] were with mixed types of cancer. Such as diversity of cancer types may have a considerable complicating effect for the interpretation of the relationships between the interventions and cytokines because of a gender difference. With regard to the duration of the cancer diagnosis, there were several criteria in each trial. Two trials [23, 32] included patients previously diagnosed with cancer for a median of 4.0, 1.1 years, respectively. In the other studies, we were unable to find a clear description; for example, one trial [38] recruited patients with “recently” diagnosed breast cancer. This heterogeneity in the duration of the cancer diagnosis may also have a significant complicating effect on the interpretation of the relationship between the interventions and cytokines. With respect to cancer stage and treatment regimen, approximately 70% of the subjects in the selected articles had Stage I or Stage II cancer, and many of the trials recruited patients with early-stage breast cancer, although one study [39] enrolled only participants with Stage III or Stage IV cancer. Treatment regimen largely depended on cancer stage. Thus, some differences were observed in treatment regimen for the study participants in each trial. One study [38] included early-stage breast cancer patients treated with breast-conserving surgery, not receiving systemic chemotherapy. Another study [40] was conducted on breast cancer survivors to examine whether there was a difference in lymphocyte recovery as a consequence of treatment regimen. Although the result was that lymphocyte recovery was generally consistent irrespective of whether chemotherapy was used in conjunction with radiation therapy, is recommended that future trials should be based on the same conditions.

There were several differences between the articles concerning the characteristics of the interventions. There were two MBSR treatment durations: either a 6- or 8-week intervention, and there was also a MAP treatment programme with a 6-week intervention. All of the MBSR programmes were based on the standard protocol produced by Kabat-Zinn [2], in which the duration of the training is set at 8 weeks. Three trials [31, 32, 38] were performed with an 8-week MBSR intervention; another two trials [39, 40] involved a 6-week MBSR; and the remainder [23] was with the described 6-week programme of MAPs. In addition, with respect to the substantive content of the MBSR programme, all but one study [39] were conducted with 1.5–2.5 h of weekly group sessions. A further-modified MBSR programme that included three in-person sessions and three audio sessions provided on CDs was also conducted for advanced-stage cancer patients [39]. This training consisted of three live sessions and three at-home practices. In line with this, future trials may require the MBI programme to be varied in accordance with patients’ conditions, specifically for advanced-stage cancer patients.

Of note was the report by Matchim et al. [13], who found that cytokine production (IFN-γ, TNF-α, and IL-4) had a large effect size (greater than 1) at 6- and 12-month follow-ups post-MBSR in early-stage cancer patients [31]. This result may indicate that further research will require longitudinal studies to detect possible effects not clearly found so far.

### Limitations

The primary limitation of this review was the small number of included studies and their different and limited quality designs, some of them with a high risk of bias, and none with a clear evaluation of the intervention integrity. Of the 13 articles, one study [31] was a 1-year follow-up trial [32], while two studies [33, 34] were conducted using the same population. Our final inclusion trials were largely based on 11 articles. As far as quality is concerned, there were only seven RCTs, including one small RCT [35] and one pilot RCT [21].

Another limitation is the biased and heterogeneous composition of the subjects. There were large bias in gender and age in healthy individuals, whereas some studies involving cancer patients were conducted using participants with different conditions, specifically with respect to the type and stage of cancer. In addition, the descriptive parameters of the groups were not shown in some studies [22, 33, 37, 38], and therefore, the recovery of specific data in order to perform meta-analytic reviews in the future will be difficult.

Additional limitations are related to methodological problems. Biomarkers were examined in diverse materials: blood, nasal secretions, blister fluid and saliva. Consequently, it is quite difficult to interpret the results even though the same biomarker was compared. There was also diversity with regard to the MBI programme frequency and duration among the trials. Although it is necessary to vary MBI training in accordance with the participant’s situation, future trials would require the use of MBI programmes with the same parameters in a more or less homogeneous population.

### Recommendations for future research

Above all, further studies should require rigorous RCT designs. With respect to the MBI protocols, they should be more homogeneous and include better descriptions if they are to enable comparisons to be made. However, the content of the MBIs could be varied depending on the participant’s situation, provided that the basic components are not changed.

In healthy individuals, the following provisions would be beneficial for the conducting of future trials: 1) the participants recruited should not only comprise older females but also young or adult males; 2) subjects with a lower BMI or increased CRP level should also be enrolled; and 3) some neuropeptides or CRP, in addition to cytokines, should also be taken into account as biomarkers [49].

Similarly, in cancer patients, these following stipulations would be of benefit: 1) the participants recruited should be homogeneous, with similar conditions, particularly with respect to cancer type, cancer stage and treatment regimen; 2) trials with a follow-up period are recommended; and 3) some neuropeptides should also be included as biomarkers.

## Conclusions

Despite a number of limitations, the results seem to indicate that MBIs have no effect on cytokines in healthy populations, probably owing to the balanced baseline levels of pro- and anti-inflammatory cytokines in these individuals. The most evident effect of MBIs in healthy persons, particularly yoga and sitting meditation, was an increase in post-intervention levels of the IGF-1 neuropeptide, an important biomarker related to cancer prevention and other clinical conditions. For NPY, despite a lack of post-intervention differences, MBIs may have a beneficial effect on enhancing recovery from stress. Finally, the results were inconsistent for CRP, most likely due to a large body mass index (BMI) interaction; MBIs might be more effective in lower-BMI individuals.

With regard to cancer patients, the conclusion of this study is that MBIs may have some effect on cytokines, although it is not possible to determine on which specific cytokines. A plausible hypothesis to be contrasted in future studies is that MBIs help the immune system to normalize, increasing the production of Th2 cytokines (e.g. IL-4), while reducing Th1 (e.g. IFN-γ) cytokines. However, given the complexity and different contexts of the immune system, and the fact that this investigation is in its preliminary stage, additional randomized controlled trials are needed to further establish the impact of MBIs programmes on biomarkers, in both clinical and non-clinical populations.

## Abbreviations

BMI: Body mass index
BSI-18: Brief symptom inventory-18
CES-D: Center for Epidemiological Studies Depression
CRP: C-reactive protein
IFN-γ: Interferon gamma
IGF-1: Insulin-like growth factor 1
IL: Interleukin
MAPs: Mindfulness awareness practices
MBCT: Mindfulness-based Cognitive Therapy
MBIs: Mindfulness-Based Interventions
MBSR: Mindfulness-Based Stress Reduction
MM: Mindfulness Meditation
MT: Mindfulness training
NK: Natural killer
NPY: Neuropeptide Y
NRCTs: Non-randomized controlled trials
PBMCs: Peripheral blood mononuclear cells
RCTs: Randomized controlled trials
Th: T-helper
TNF-α: Tumour necrosis factor alpha
